# A food hub to address healthy food access gaps: Residents’ preferences

**DOI:** 10.5304/jafscd.2019.091.010

**Published:** 2019-05-01

**Authors:** Jill K. Clark, Chaturia Rouse, Ashwini R. Sehgal, Mary Bailey, Bethany A. Bell, Stephanie N. Pike, Patricia A. Sharpe, Darcy A. Freedman

**Affiliations:** a John Glenn College of Public Affairs; Ohio State University; 1810 College Road; Columbus, OH 43210 USA; b Prevention Research Center for Healthy Neighborhoods, Population and Quantitative Health Sciences, Case Western, Reserve University, School of Medicine; c Center for Reducing Health Disparities, Case Western Reserve University; d College of Social Work, University of South Carolina; e Prevention Research Center, Arnold School of Public Health, University of South Carolina

**Keywords:** Food Hub, Food Access, Food Desert, Urban, Poverty, Consumer Demographics, Consumer Preferences, Food Environment

## Abstract

Interventions aimed at improving access to healthy food in low-income communities should consider the preferences of residents. Household food shoppers in two urban, low-income communities were asked about their preferences for vendors at, and qualities of, a potential nearby food hub. Universally, participants preferred availability of whole foods, primarily fruits and vegetables. They also favored cleanliness, quality, and affordability. The demographics and preferences of potential customers raise central issues that would need to be integrated into the development of a food hub, namely affordability (likely through subsidization), attention to accommodation and cultural accessibility, and programming that builds community.

## Introduction

Low-income communities often have less physical access to affordable, healthy foods because of a lack of supermarkets and supercenters. Although referred to as ‘food deserts,’^1^ these areas often have food retail shopping options in the form of convenience stores, resulting in higher prices and a lower quantity and quality of healthy items (Beaulac, Kristjansson, & Cummins, 2009; Walker, Keane, & Burke, 2010). Areas that lack healthy food options are disproportionately communities of color experiencing high rates of poverty; these communities are also associated with relatively higher levels of poor mental and physical health outcomes, such as greater levels of stress, poorer diet quality, and greater food insecurity (Caspi, Sorensen, Subramanian, & Kawachi, 2012; Clifton, 2004; Ver Ploeg, 2012; Walker et al., 2010). Due to these disparities, the implementation of place-based strategies that address inequities in the food environment is now a focus of many federal, state, and local policy initiatives.

One place-based strategy is a food hub. Food hubs are growing in popularity in the United States as a way to address shortcomings of the conventional food system (Colasanti, Hardy, Farbman, Pirog, Fisk, & Hamm, 2018; Levkoe et al., 2018). The most common shortcoming addressed by food hubs is a lack of market access and growth opportunities for small and mid-size producers (Woods, Velandia, Holcomb, Dunning, & Bendfeldt, 2013). More than 80% of all food hubs have this producer-focused goal (Colasanti et al., 2018). As such, food hubs are often described as centers for aggregating, distributing, and selling locally or regionally sourced foods from multiple producers to access and expand markets and meet the demands of buyers (Woods, Velandia, Holcomb, Dunning, & Bendfeldt, 2013). In addition, some food hubs are emerging to address shortcomings of the food system from the consumer perspective, such as a lack of access to healthy food in certain communities (Colasanti et al., 2018; Levkoe et al., 2018; Koch & Hamm, 2015). For instance, food hubs with this goal are offering a wide range of educational services, such as nutrition and cooking classes; they also may have features such as a small grocery store or mobile market and accept Supplemental Nutrition Assistance Program (SNAP) benefits to increase affordability (Colasanti et al., 2018; Koch & Hamm, 2015). Food hubs also have goals that, while not directly involving food, aim to positively transform the food system; these goals include fostering local decision-making power, keeping money within the community, providing a venue for entrepreneurship and new jobs, and serving as a vehicle for community-based economic development (Levkoe et al., 2018; Lerman, 2012; Matson & Thayer, 2013).

While the USDA provides a definition for food hubs (Barham, Tropp, Enterline, Farbman, Fisk, & Kiraly, 2012), the range of purposes for food hubs is likely the reason why no single definition of a food hub is universally accepted. In practice, food hubs have varying missions, values, business models, features, services, and customer bases (e.g., institutional, other retail, and consumers) (Horst, Ringstrom, Tyman, Ward, Werner, & Born, 2011; Levkoe et al, 2018). They emerge from varying contexts and are designed to meet needs of a community rooted in a particular place, giving each food hub a unique expression (Levkoe et al., 2012).

Embedded in this flexibility are practical tensions, particularly for food hubs that have non-financial goals strongly related to their mission, such as increasing healthy food access for low-income consumers. These hubs often find that they are pulled in different directions by competing forces, such as the need to be viable in a market economy and the desire to be agents of social justice (Levkoe et al., 2018). For example, in areas that lack access to healthy foods, the aggregation of product can also be a strategy to reduce costs to low-income consumers. Yet, given that most food hubs aim to increase the viability of small and mid-size producers, the aggregation of source-identified local product, on one hand, can increase the price premiums for local producers; this can, in turn, produce a tension between the needs of the producer and the needs of low-income consumers. Indeed, Koch and Hamm (2015) found that despite the fact that many food hubs in the Mid-west aimed to increase healthy food access for underserved customers, maintaining a viable food hub business was the first priority of hub management. Another tension for social enterprises^2^ is the need to fund social and community-based services, likely creating a reliance on grants, which impacts long-term sustainability of the business (Colasanti et al., 2018; Levkoe et al., 2018).

Despite these tensions, in 2014, a local community development corporation in Cleveland, Ohio, saw this flexibility in food hub definition as an opportunity to link goals for improving healthy food access, economic development, and agricultural and culinary job opportunities in an area with a high rate of poverty, food insecurity, and limited access to healthy food retailers. The community development corporation received a Healthy Food Financing Initiative grant to support the development of a food hub, a term adopted by the development corporation to describe their work. This food hub was conceptualized as having the following goals: (1) create employment and business opportunities; (2) bring healthy, local, affordable food choices to high need communities; (3) develop a healthy food distribution system; and (4) implement strategies that promote and encourage healthy food education and consumption.

In this research, we sought to examine the consumer preferences for this new food hub. We found little market research targeting residents of so-called food deserts, despite nearly 50% of food hubs actively operating in such places, 43% of food hubs accepting SNAP benefits, up to 37% of hubs organizing around a direct-to-consumer model, and 68% of food hubs having at least some direct-to-consumer sales (Barham et al., 2012; Colasanti et al., 2017; Feldstein & Barham, 2017).^3^ A notable exception, however, is Weatherspoon, Oehmke, Coleman, Dembele, and Weatherspoon’s (2012) study in which they examined the preferences of consumers from food deserts for specific fruits and vegetables, income and price responsiveness, and the many constraints low-income consumers face in general (not in regards to a food hub). They found that consumers from food deserts respond to the introduction of fresh fruits and vegetables in the neighborhood; however, they also found that lower purchasing power would need to be coupled with incentives to increase purchasing.

The purpose of this research brief is to inform the development of food hubs that seek to increase access to healthy foods for low-income residents. Our goal was to examine the following research questions based on resident surveys collected prior to opening the food hub: (1) How important are specific food hub vendors and food hub qualities to potential customers? and (2) Do these preferences vary depending on how likely customers are to shop at the food hub?

## Study Sites, Survey, Methods

This research emerged from a natural experiment in which a food hub was being developed and implemented in Cleveland, Ohio, under the direction of a local community development corporation and their partners. A separate research grant was awarded to external evaluators to evaluate the impact of the food hub on resident norms and dietary behaviors using a quasi-experimental design involving residents in the Cleveland community and a comparison group from a socio-demographically similar community in Columbus, Ohio. These two communities are located approximately 150 miles (241 km) apart. Both communities were classified as urban food deserts, having both low access to a full-service supermarket within one mile (1.6 km) and being low income (Pike et al., 2017). The present analysis focuses on participants from both communities prior to developing the food hub with the goal that our findings will provide guidance to other communities implementing food hubs as a strategy to promote healthy food access.

Participants included 482 household food shoppers who completed a baseline phone survey between August 2015 and September 2016, providing answers to all questions relating to demographics, their current health condition, their food hub vendor and quality preference, and their likely shopping frequency.^4^ Table 1 describes the demographics of the participants. A little over two-thirds of participants are African-American and female with a high school level of education or less. Sixty-one percent are not employed. Household income is low, with most reporting an annual income of less than US$20,000 and 64.5% reporting the receipt of SNAP benefits within the last 12 months. A little over half do not use their own cars for their main grocery shopping. The majority of participants self-reported a diagnosis of high blood pressure, heart disease, diabetes, and/or obesity.

Given that no food hubs existed near these communities and the developers of the food hubs considered a “hub” to be a new and evolving (and flexible) concept, we asked respondents to “imagine” one. Further, because we asked for feedback on something that was novel, we did not want to limit their imagination. We informed participants that the hub could have multiple farmers or vendors selling a wide variety of foods in the same area, which may also have restaurants. Further, we explained that, unlike a farmers market, the food hub would be open seven days a week.

Participants used a 4-point Likert scale when stating how important particular vendors would be in determining whether to shop at a neighborhood food hub. Responses ranged from 1=‘Not at all important’ to 4=‘Very important,’ and they included the option ‘I would not shop at the food hub.’ Additionally, their interest level in being a vendor or employee at the food hub was assessed with the 3-point Likert scale ranging from 1=‘Not at all interested’ to 3=‘Very interested.’

To analyze the preference data, we first calculated the mean for vendor and hub quality preferences across participants. Significant differences between each mean preferences (*p*<.05) were then tested using repeated measures ANOVA with post-hoc tests for pair-wise comparison using the Bonferroni correction (i.e., comparing each vendor preference with all other vendor preferences and each hub quality preference with all other hub quality preferences). Next, these preferences were examined by the likely frequency of shopping. Respondents were asked how frequently they currently shop at their main food shopping locations and then how frequently they would shop at a neighborhood food hub. These two frequencies were used to create a new variable indicating whether participants were likely to shop at the food hub at more, less, or about the same frequency as their main shopping locations. For each food hub vendor and hub quality, significant differences between food hub shopping frequencies were tested using one-way ANOVA (*p*<.05) with post-hoc tests. All analyses were performed using SPSS, version 24.

## Preferences for Vendors and Food Hub Qualities

Tables 2 and 3 rank order the mean importance of specific food hub vendors and qualities across all participants. In Table 2, similar vendors are grouped and then ranked from highest to lowest by the mean preference score across participants. Overall, preferences for vendors are significantly different (*F*_11,471_ = 79.211, *p*<.001). To examine the pair-wise differences, Table 2 includes cross-listings of each vendor type. On the right side of the table an “X” denotes a significant difference between the vendor listed on the left and the vendor titled in that column with an “X.” For example, preferences for fruit and vegetable vendors are significantly different than preferences for all other vendors. Preferences for cheese and dairy vendors are significantly different than preferences for all other vendor *except* for fish or seafood vendors. Preferences for cheese and dairy are not significantly different than fish or seafood.

Participants ranked fruit and vegetable vendors highest, followed by meat vendors, both of which are significantly more important than any other food vendor types listed. Vendors selling whole foods such as fruits, vegetables, meats, and fish were rated as being more important than vendors selling nonperishable foods, ready to eat foods, and nonfoods such as flowers.

Table 3 presents the preferences for qualities of a food hub. Like preferences for vendors, overall, preferences for food hub qualities are significantly different (*F*_10,472_ = 120.502, *p<*.001). To examine pair-wise differences, Table 3 is read the same as Table 2. On the right side of the table an “X” denotes a significant difference between the vendor listed on the left and the vendor titled in that column with an “X. Customer service qualities were rated as the most important features of a food hub. Cleanliness was rated the highest across all participants, followed by quality. While cleanliness and good quality are significantly different from all other qualities, they are not significantly different from one another. Affordability is ranked third and is significantly higher than the other categories of ease of use, community engagement, and employment opportunities. The ease of use category was the next most important (although not significantly different than welcoming and variety), indicating a preference for a food hub that is convenient and offers one-stop shopping. The community engagement category was significantly less important, indicating lower preferences for having vendors and customers from the community. Finally, the least important qualities related to employment at the food hub. While being a vendor at the food hub or an employee at the hub ranked at the bottom, it should be noted that 78% of respondents are at least some-what interested in employment or vending opportunities (responding with a 2 or 3 on the 3-point Likert scale).

Tables 4 and 5 show food hub vendor and quality preferences by intended food hub shopping frequency. Almost half of the participants (47.5%) reported that they would shop at a food hub less frequently than their main stores. Overall, few significant differences exist between intended food hub shopping frequency and preferences, which include preferences for vendors (Table 4). Participants that intend on shopping at the same or greater frequency at the hub prefer fruits and vegetables, herbs and spices, general merchandise, and staple goods more than those who intend to shop less frequently. Staples and general merchandise preferences seem logical given that these items are part of the full shopping experience that is associated with their main food shopping location (supermarket or supercenter). Likewise, variety and convenience are significantly more important to more frequent shoppers (Table 5). Finally, being interested in vending or working at the food hub is significantly correlated with intended shopping frequency at the food hub.

## Considerations when Intervening in Food Deserts

Among this sample of low-income urban residents, participants expressed preferences for access to whole foods from a food hub, particularly fruits and vegetables. This aligns with typical foods offered at food hubs and the types of foods aimed at increasing healthy food access and improved nutrition (Colasanti et al., 2018). Preferences for prepared foods ranked very low, which could be related to the fact that these products are ineligible for purchase using SNAP benefits. Considering that 65.5% of the sample were SNAP recipients, the ineligibility of purchasing prepared foods with SNAP benefits could very well be a contributing factor their low ranking among participants. Coupling food hub implementation with programming to further demand for whole foods, such as community cooking and nutrition classes (Hardy, Hamm, Pirog, Fisk, Farbman, & Fischer, 2016) or other programs aimed at reducing the costs of whole foods (such as the USDA Food Insecurity Incentive Program), may increase the financial sustainability of food hubs in healthy food access gap contexts.

Cleanliness, product quality, and affordability were the most preferred qualities of the food hub environment. The salience of cleanliness for a food hub could suggest that food retail spaces in low-income neighborhoods may inconsistently meet desired cleanliness standards. These preferences may indicate a need for place-based food environment interventions to be designed to accommodate community needs and preferences, in addition to physical and economic access to healthy foods. Food environment interventions are critiqued for not being ‘for’ specific neighborhoods. For example, farmers market interventions are described as already ‘raced,’ ‘classed,’ and ‘othering’ by residents from low-income neighborhoods (Larchet, 2014). In this research, we asked participants with little prior knowledge of what a food hub is to “imagine” a food hub in their neighborhood. This intervention may have been perceived to be more inclusive and better aligned with shopping preferences. Future research will explore how these perceptions change once a food hub is realized in a neighborhood.

While still ranking as important, on average, when asked about preferences for vendors and customers being from the community, these categories ranked lower than the food hub environment and product features. To address the underlying relational- and values-based principles of a food hub, developers in a food desert will likely need to consider designing programming to build local residents’ commitment to engage with the hub. Moreover, developers should consider thinking more broadly about market research and include other dimensions beyond preferences to determine community readiness for a food environment intervention (Edwards, Jumper-Thurman, Plested, Oetting, & Swanson, 2000).

The socio-demographics of the target and control communities may give food hub developers pause. Concerns regarding market viability exist when purchasing power is low and unemployment is high (Ver Ploeg, Dutko, & Breneman, 2014). Given the rates of SNAP participation, integrating the ability to pay with SNAP benefits would help ensure both economic and sociocultural accessibility. Low purchasing power raises the tension between market-driven and justice-driven approaches to food hub development and signals the need for a social enterprise to make products more affordable (via, for example, grant dollars or public-private partnership) and to sustain the market.

Finally, an issue raised in these data is the opportunity to address two community needs simultaneously: improving healthy food access and providing employment opportunities. A food hub has the potential to accomplish both goals if both are prioritized. Findings from this research, given that participants reported high rates of unemployment and a desire to work at a food hub, reveal the need for food hub interventions to begin with a dual focus on healthy food access and employment.

## Conclusion

Areas that lack healthy food access represent a shortcoming of the conventional food system. Bringing a market-based solution, like a food hub, to such areas—ones that supermarkets have largely avoided—is filled with tensions. However, existing hubs demonstrate that food hubs can be flexible entities that can and, in some cases, are run as social enterprises. To address these tensions, food hub developers aiming to serve low-income residents in an area that lacks access to healthy food can take the first step by understanding market demand—i.e., understanding their customer profile, their customer preferences for specific products (e.g., specific fruits and vegetables, rather than the broad product category of “fruits and vegetables”), and understanding preferences for the quality of the shopping environment. A next step would be to determine community readiness and any needed programming to accompany the market component of the hub.

In our study, participants living in two food deserts were surveyed and asked to imagine a neighborhood food hub and state their preferences for food hub vendors and hub qualities. Their whole food preferences align with typical food hub vendors. However, the demographics and preferences of potential customers also raise central issues that would need to be integrated into the development of a food hub, namely affordability. This can likely be accomplished through subsidization, attention to accommodation and cultural accessibility, conjoint programming that builds community and commitment, and jobs training and employment. These types of developments would be handled best by a social enterprise oriented toward community development.

## Figures and Tables

**Table 1.** Participant Demographics (*N*=482)

Demographics	Participants (#)	Percent^a^ or Mean
**Gender**		
Female	354	73.4%
Male	128	26.6%

**Education**		
Grades 1–11	103	21.4%
Grade 12 or GED	186	38.6%
Technical School or Some College	134	27.8%
College Graduate	58	12.0%

**Employment**		
Employed	188	39.0%
Out of Work	94	19.5%
Not in Workforce^b^	199	41.2%

**Household Size (Average)**	2.56	

**Household Income ($US)**		
Less than 10,000	176	36.5%
$10,001–$20,000	146	30.3%
$20,001–$30,000	76	15.8%
$30,001 or More	78	16.2%

**Supplemental Nutrition Assistance Program**		
SNAP Participant	311	64.5%
Non-participant	170	35.3%

**Race**		
White	130	27.0%
Black or African American	332	69.0%
Other	19	4.0%

**Car Use for Main Food Shopping**		
Use Own Car	255	52.9%
Do Not Use Own Car	226	46.9%

**Self-reported Diagnosed Health Condition**		
High Blood Pressure	195	40.5%
Heart Disease	37	7.7%
Diabetes	76	15.8%
Obesity	139	28.8%
One of the Above Conditions	268	55.6%

a Calculations exclude missing data.

b Choices were Unable to Work, Homemaker, Student, and Retired.

**Table 2.** Participants’ Food Hub Vendor Preferences

Food Hub Vendor Type	Mean	Std. Dev.	Fruit and Vegetable	Fresh Meat or Butcher	Cheese and Dairy	Fish or Seafood	Herbs and Spices	Pasta and Dry Goods	Bakery	Staple Goods	Value-added	Prepared Foods	General Merchandise	Fresh Cut Flowers or Plants
**Vendors of Whole Food Products**						**Significantly Different from *p*<.05**								

Fruit and Vegetable	3.81	0.45		X	X	X	X	X	X	X	X	X	X	X
Fresh Meat or Butcher	3.71	0.6	X		X	X	X	X	X	X	X	X	X	X
Cheese and Dairy	3.55	0.68	X	X			X	X	X	X	X	X	X	X
Fish or Seafood	3.54	0.82	X	X			X	X	X	X	X	X	X	X
Herbs and Spices	3.34	0.8	X	X	X	X			X	X	X	X		X

**Vendors of Nonperishables**														

Pasta and Dry Goods (e.g., beans, grains)	3.27	0.78	X	X	X	X			X	X	X	X		X
Bakery	3.06	0.84	X	X	X	X	X	X				X	X	X
Staple Goods (e.g., coffee, flour, sugar)	3.01	0.95	X	X	X	X	X	X			X		X	X

**Vendors of Ready-to-Eat Foods**														

Value-added (e.g., pre-cut fruit, salsa, jam)	2.96	0.89	X	X	X	X	X	X				X	X	X
Prepared Foods	2.73	0.97	X	X	X	X	X	X	X	X	X		X	

**Vendors of Nonfoods**														

General Merchandise (e.g., toiletries, diapers)	3.32	0.96	X	X	X	X			X	X	X	X		X
Fresh-cut Flowers or Plants	2.66	0.99	X	X	X	X	X	X	X	X	X		X	

**Table 3.** Participants’ Food Hub Quality Preferences

	Mean	Std. Dev.	Clean	Good Quality	Affordability	People Are Welcoming	Good Variety	Convenient to Shop	One-stop Shop	Vendors from Comm.	Customers from Comm.	Work as an Employee	Work as a Vendor
**Customer Service and Product Qualities**					**Significantly Different from *p*<.05**								

Clean	3.95	0.24			X	X	X	X	X	X	X	X	X
Good Quality	3.91	0.33			X	X	X	X	X	X	X	X	X
Affordability	3.85	0.44	X	X	X			X	X	X	X	X	X
People Are Welcoming	3.78	0.48	X	X						X	X	X	X
Good Variety	3.77	0.49	X	X						X	X	X	X

**Ease of Use**													

Convenient to Shop	3.73	0.54	X	X	X					X	X	X	X
One-stop Shop	3.69	0.63	X	X	X					X	X	X	X

**Community Engagement**													

Vendors Are from Community	3.1	0.87	X	X	X	X	X	X	X			X	X
Customers Are from Community	3.01	0.92	X	X	X	X	X	X	X			X	X

**Employment Opportunities**													

Work as an Employee	2.23	0.04	X	X	X	X	X	X	X	X	X		X
Work as a Vendor	1.91	0.04	X	X	X	X	X	X	X	X	X	X	

**Table 4.** Mean Food Hub Vendor Preferences by Food Hub Shopping Frequency

	Intended Food Hub Shopping Frequency Compared to Main Store		

Food Hub Vendors	Less Frequently	About the Same	More Frequently
**Vendors of Whole Food Products**			

Fruit and Vegetable	3.77	3.85	3.87
Fresh Meat or Butcher	3.66	3.71	3.79
Cheese and Dairy	3.51	3.54	3.65
Fish or Seafood	3.56	3.46	3.65
Herbs and Spices	3.24	3.42	3.42

**Vendors of Nonperishables**			

Pasta and Dry Goods (e.g., beans, grains)	3.25	3.29	3.3
Bakery	3.03	3.04	3.16
Staple goods (e.g., coffee, flour, sugar)*	2.96	2.97	3.23

**Vendors of Ready-to-Eat Food**			

Value-added (e.g., pre-cut fruit, salsa, jam)	2.91	2.98	3.01
Prepared Foods	2.71	2.80	2.7

**Vendors of Nonfoods**			

General Merchandise (e.g., toiletries, diapers)*	3.21	3.40	3.47
Fresh Cut Flowers or Plants	2.60	2.68	2.77

*N*=	229	142	111

* significant at *p*<.05

**Table 5.** Mean Food Hub Quality Preferences by Food Hub Shopping Frequency

	Intended Food Hub Shopping Frequency Compared to Main Store		

Food Hub Qualities	Less Frequently	About the Same	More Frequently
**Customer Service and Product Qualities**			

Clean	3.95	3.97	3.94
Good Quality	3.89	3.94	3.95
Affordability	3.81	3.86	3.91
People Are Welcoming	3.75	3.82	3.81
Good Variety*	3.74	3.78	3.86

**Ease of Use**			

Convenient to Shop	3.69	3.77	3.81
One-stop Shop	3.62	3.71	3.75

**Community Engagement**			

Vendors Are from Community	3.07	3.19	3.08
Customers Are from Community	2.95	3.12	3.02

**Employment Opportunities**			

Work as an Employee*	2.13	2.19	2.47
Work as a Vendor*	1.80	1.97	2.08

N=	229	142	111

* significant at *p*<.05

## Footnotes

1 Areas with no supermarkets, but with fast food and convenience options are often referred to as “food swamps” (Rose, Bodor, Swalm, Rice, Farley, & Hutchinson, 2009). Our team prefers to use the term “healthy food access gaps” instead of “food deserts” or “food swamps.” Food deserts suggest a lack of food, when these locations, particularly urban locations, often have a plethora of unhealthy food (Pike et al., 2017). Further, the terms “desert” and “swamps” are not asset-based approaches to characterizing community members’ residential locations.

2 A social enterprise can be defined as a nongovernmental organization (e.g., private nonprofit or for-profit) using a market-based approach to solving social problems (Kerlin, 2006).

3 Food deserts, as defined by the Food, Conservation, and Energy Act of 2008, are areas “with limited access to affordable and nutritious food, particularly such an area composed of predominantly lower-income neighborhoods and communities.”

4 The full study has 519 participants. For this paper, we limited it to the 482 participants who completed all sections of the survey needed to complete the analysis.
